# Perspectives on Virtual Care for Childhood Cancer Survivors in Non-Metropolitan Areas during the COVID-19 Pandemic

**DOI:** 10.3390/curroncol30090588

**Published:** 2023-09-01

**Authors:** Rachel Phelan, Taiwo Opeyemi Aremu, Jeffrey Karst, Lynnette Anderson, Anna Jordan, Jocelyn Morin, Julie Nichols, Ashima Singh, Debra Schmidt, Jennifer A. Hoag, Char Napurski, Haley Zweber, Karim Thomas Sadak

**Affiliations:** 1 Department of Pediatrics, Medical College of Wisconsin, 8701 Watertown Plank Road, Milwaukee, WI 53226, USA; rphelan@mcw.edu (R.P.); jkarst@mcw.edu (J.K.); landerson@chw.org (L.A.); anjordan@mcw.edu (A.J.); ashimasingh@mcw.edu (A.S.); jhoag@mcw.edu (J.A.H.); 2 University of Minnesota Masonic Children’s Hospital, University of Minnesota Masonic Cancer Center, 420 Delaware St. SE—Mayo MMC 484, Minneapolis, MN 55455, USA; aremu006@umn.edu (T.O.A.); bake0257@umn.edu (C.N.); zwebe060@umn.edu (H.Z.); 3 Children’s Wisconsin, 9000 W. Wisconsin Avenue, Milwaukee, WI 53226, USA; jmorin@chw.org (J.M.); janichols@chw.org (J.N.); dschmidt@chw.org (D.S.)

**Keywords:** telemedicine, COVID-19 pandemic, long-term follow-up, childhood cancer survivors, rural health

## Abstract

The COVID-19 pandemic paved the way for the widespread use of virtual care for childhood cancer survivors (CCSs). CCSs were virtual recipients of diverse care, including long-term follow-up (LTFU), primary care, mental health care, and several others. Virtual care comes with well-documented benefits and challenges. These are further magnified for CCSs living in rural or non-metropolitan areas. Here, we describe the virtual care of CCSs from two Upper Midwest cities with well-established childhood cancer survivor programs within large comprehensive cancer centers in the United States. CCSs from non-metropolitan areas, especially CCSs with two or more late effects, used virtual care more often during the COVID-19 pandemic compared to CCSs from metropolitan areas. A review of the related literature is also included and the identified challenges in providing virtual care, such as privacy concerns, technology-connectivity constraints, and medical license restrictions. Despite these limitations, the care of CCSs has evolved to leverage virtual care and its ability to increase access for patients and promote continuity of care for CCSs living in rural areas.

## 1. Introduction

Virtual care, also known as telemedicine or telehealth, refers to the use of technology to provide remote healthcare services. It involves the delivery of medical consultations, diagnoses, treatments, and monitoring using various communication platforms such as video calls, phone calls, secure messaging, and remote monitoring devices. It can be of great utility if carried out in a way that is accessible, reliable, and meets the goals of the patients, providers, healthcare systems, and payers. Virtual care has become increasingly popular and important, especially in situations where in-person visits are not easily accessible or convenient. Amid the COVID-19 pandemic, pediatric oncology clinics had to transition to a predominantly virtual model, including for cancer survivorship care [1]. Multiple modalities were and continue to be utilized, including phone visits, video visits, online websites, smartphone applications, and email communication [2]. The foundation of cancer survivorship care is health promotion and late-effects screening for childhood cancer survivors (CCSs). Like all medical care, the COVID-19 pandemic resulted in secondary concerns such as limited access to care and detrimental effects on the psychological and social well-being of CCSs. While the transition from in-person to virtual care has not been without difficulties, the increased use of technology has shown promise in working both as a substitute for in-person care and as an adjunct to in-person visits [3]. This is especially true for survivors in rural settings with traditionally limited access to large metropolitan area health systems with cancer centers and designated survivor-focused care teams [4].

While the change from in-person visits to virtual care initially occurred quickly for healthcare systems, providers, and patients, it is unclear how this care has evolved for CCSs living in rural settings. Virtual care has expanded its footprint to include long-term follow-up (LTFU) visits, primary care, counseling and mental health care/support, health coaching, integrative medicine care, rehabilitation medicine, remote monitoring, health education, and many others. In some settings with virtual care, survivors report positive experiences, noting that the virtual option provides many survivors with improved access to care [5].

## 2. Objective

This short report aims to highlight the advantages of using virtual care for CCSs in the Upper Midwest region of the United States and its potential benefits for CCSs living in rural communities.

## 3. Perspectives on the Advantages and Disadvantages of Virtual Care for CCS

Many CCSs live far from their treating institution or live in rural areas without access to specialists who have the expertise to recommend late-effects screening and management. This distance from the treating institution can be a barrier for survivors, who may need to miss school or work to attend LTFU appointments. They may also have difficulty coordinating multiple appointments on the same day. They may additionally face the financial burden of medical expenses and travel for care. Virtual care can help minimize the time missed at school or work to attend these appointments but providing a more streamlined experience to meet with a team of professionals with the unique late-effects knowledge of related risks for the individual CCS. Virtual care also has the advantage of the patient and families not being required to travel and may ease many of the aforementioned logistical burdens. This can significantly reduce the financial burden and stress on childhood cancer survivors and their families, making healthcare more accessible and affordable. It is especially beneficial for survivors who may have mobility limitations or ongoing treatment-related issues. Virtual care offers LTFU providers the ability to counsel, review screening tests, provide recommendations, and order tests and referrals to ensure survivors receive the necessary risk-stratified care. The use of technology may decrease the time and financial burden of LTFU care and offer alternatives that promote long-term relationships with experts in late-effects care. As CCSs transition into adulthood, having a relationship with medical professionals who understand their treatment as children and the impact on their lives as they age may help to ensure that survivors receive the life-long screening they need and early detection and management of late effects. Virtual care enhances this process by enabling survivors to maintain regular contact with their healthcare providers thereby ensuring continuity of care. Virtual-care platforms can facilitate care coordination among multiple healthcare providers involved in a childhood cancer survivor’s care. Providers can easily share medical records, collaborate on treatment plans, and communicate effectively, ensuring a holistic and coordinated approach to healthcare. Virtual platforms can connect childhood cancer survivors with other survivors, support groups, and online communities, fostering peer support and shared experiences. This can be particularly valuable for survivors in rural communities who may have limited local support networks. Virtual care facilitates ongoing surveillance of survivors’ health, minimizing the risk of long-term complications going unnoticed. In a nutshell, virtual care has the potential to improve access to specialized care, support survivorship needs, and enhance the overall quality of life for childhood cancer survivors in rural communities. It empowers survivors to actively participate in their healthcare journey while minimizing the barriers they may face in accessing necessary care and support.

Virtual care can result in a financial burden for patients who must finance their internet connectivity themselves. Virtual care also comes with the challenges of incomplete physical examinations paving the way for misdiagnosis or the need for additional in-person appointments. The increased need for care-coordination support has been noted with virtual care, as patients are unable to get laboratory tests or complete screening tests at the same time as their visits, which would often be the case through in-person visits. The resulting care can become fragmented and medical information then needs to be collected across multiple sites or health systems. Ultimately, there is also concern that the provider–patient relationship cannot be developed or strengthened effectively through virtual care. In addition, for those patients that prefer in-person care, virtual care could represent a clear dissatisfier.

## 4. Real World Experience with Virtual Care for CCSs

In a program-evaluation effort, descriptive information during the early phases of the pandemic was collected on the virtual care of CCSs living outside of metropolitan areas. These CCSs received care in two large metropolitan Upper Midwest cities with comprehensive cancer centers and specialized childhood cancer survivor programs. Metropolitan areas were defined by US Census Bureau zip codes and used instead of the rural vs. non-rural distinction, as almost all localities were considered rural in these two Upper Midwest states. Most of the virtual visits included CCSs from metropolitan regions (62%, 137/221). The same was true for in-person visits, but to a larger degree (82%, 404/493). For CCSs from non-metropolitan regions, visit types were evenly split, 51% (89/173) in person and 49% (84/173) virtual. For metropolitan CCSs, most visits were in person (75% in person vs. 25% virtual). These comparisons suggested that more CCSs from non-metropolitan areas utilized virtual care than CCSs from metropolitan areas in these two cities. When this comparison was examined in survivors with two or more late effects, 30% (130/437) of all visits were from CCSs living in non-metropolitan areas (Table 1), whereas for all CCSs, 24% (173/714) were from non-metropolitan areas (Table 2). For these same survivors with two or more late effects, 37% (160/437) of visits were virtual, while 31% (221/714) were virtual for all CCSs. In summary, a greater percentage of CCSs from non-metropolitan areas were utilizing virtual care compared to CCSs from metropolitan areas (49% vs. 25%), and this trend continued when examining CCSs with two or more late effects compared to all CCSs accessing virtual care (37% vs. 31%).

A homogeneity test showed a large chi-squared value (x^2^ = 72.58, 1DF) signaling a highly significant association between the type of visit and the residence/location of patients (*p* < 0.0001). Furthermore, the odds of a virtual visit taking place among survivors that reside in a non-metro area is 5.6 (OR = 5.61; 95% CI, 3.90–8.51). This finding is consistent with our experience when considering the visit of choice for our patients during the COVID-19 pandemic.

Telemedicine provides a unique opportunity to address the psychosocial challenges of pediatric cancer survivors as well. Telemedicine has been shown to be feasible and acceptable in pediatric and adolescent and young adult (AYA) cancer patients [6,7]. It can reassure pediatric patients with cancer and survivors through reliable access to care and continuity of care for emotional health needs [8,9]. Various technological modalities—especially social media through blogs, messaging, narrative videos, and more—have created a community for the CCSs, providing social support to those who may not receive it from their own local in-person social network [2].

Telemedicine can present logistical challenges, including privacy issues at home, internet connectivity issues, and restrictions on professional licensing between states. However, physician concerns primarily revolve around the proper function of technology, the technological literacy of patients, providers, and staff; and restrictions on technology-only visits that lack access to labs, imaging, and physical exams [5]. Studies have shown that many technological concerns decrease over time as users become more familiar with the technologies being utilized [5].

There is a concern that virtual care can fragment utilization patterns and dramatically increase the need for care coordination. If survivor-focused care occurs virtually, strong partnerships with local providers, especially primary care providers, will be necessary and critical. Through these relationships with the medical homes of CCSs, many diagnostic tests and screening/surveillance testing can take place locally within their community. However, coordinating these tests locally requires intensive communication and partnership between the survivorship specialist and the local provider. In many centers, the role of nurse care coordinators or patient navigators has successfully taken on this increased amount of care coordination needs, helping identify the necessary skills that personnel must possess to overcome this barrier to care and the resulting burden on survivorship care teams.

## 5. Virtual Care for CCSs in Rural Communities

Utilizing virtual-care models may also be especially important in adolescent survivors living in rural settings. This population faces additional challenges, and they eventually need to increase their knowledge of the medical system and their medical history as they transition to being in charge of their healthcare as young adults [4,10]. The majority of the AYA population have proficient technical skills, growing up in technology-heavy environments, and when given the opportunity to receive care through telemedicine, the majority prefer online visits and find them as helpful or nearly as beneficial as their in-person visits [2,5,11]. Furthermore, many patients have felt that communication improved with their providers during virtual visits [10]. The majority shared that these visits could be a substitute for most, if not all, of their regular LTFU visits [5,10]. Care providers shared similar sentiments, with the majority considering virtual visits an adequate substitute for in-person visits [5].

There is also evidence to support the use of mobile health interventions for adolescent-aged CCSs, including promising data on acceptability and feasibility [12]. While there are clear differences between telemedicine and many mobile health interventions, both share the opportunity to promote health-related knowledge and survivor-focused care. Leveraging technology to increase survivor engagement is critical, and having implementation science at the forefront of related research will be necessary to ensure improvements in care for CCSs of rural communities. Although virtual care can bridge the geographical gap and provide childhood cancer survivors in rural communities with access to necessary healthcare services, support, and resources, it is important to note that certain aspects of care may still require in-person visits for physical examinations or diagnostic tests. Therefore, a hybrid approach that combines virtual care with periodic in-person appointments may be the most effective way to provide comprehensive care to childhood cancer survivors in rural areas.

## 6. Perspectives and Avenues for Future Research

In-depth interviews or focus groups with CCSs living in remote locations might give useful insights into their experiences with virtual care. Understanding the rewards and obstacles people encountered during the epidemic and how this affected their entire care journey can help improve virtual-care tactics.

Addressing privacy issues has surfaced as a barrier to providing virtual care. Further study might investigate specific privacy challenges confronting CCSs and propose solutions to improve data security and confidentiality in virtual-care environments.

Interventional research geared towards improving equitable access to virtual care services can be developed to promote health equity in terms of care utilization among CCSs from various socioeconomic backgrounds.

To adapt virtual care to the COVID-19 postpandemic era, it could be worthwhile to investigate how the use of virtual care for CCSs changed, considering both its ongoing usage and the reintegration of in-person care. Research can also focus on examining if virtual care can be incorporated into standard survivorship care practices.

It will be resourceful to investigate the long-term impact and sustainability of virtual care models. Research can focus on understanding the scalability, adoption, and long-term viability of virtual care approaches, considering factors such as reimbursement models, policy implications, and organizational readiness.

As technology improves and expands the quality of virtual care, it will be worthwhile to investigate the use of remote monitoring devices, wearable technology, and sensors in virtual care. Research can explore the effectiveness of these tools in monitoring vital signs, symptom tracking, medication adherence, and early detection of complications.

It will also be critical to prioritize and evaluate that virtual survivorship care does not widen health care disparities for non-metropolitan or rural CCSs. Instead, it should be an avenue to more equitably engage CCSs living in rural settings and deliver the necessary LTFU care. While this is expected to enhance the efficacy, accessibility, and impact of virtual care, it will also pave the way for a more patient-centered, efficient, and inclusive healthcare system.

To further enhance virtual care, future studies can be designed to analyze the CCSs’ satisfaction with virtual care and its influence on their entire healthcare experience. This could give insight into some of the elements influencing patient experiences.

## 7. Conclusions

Access to childhood cancer survivorship care for patients in non-metropolitan settings is still under-studied, and the impact of the transition to virtual care has not been fully examined. This study provides an insightful analysis of virtual care for pediatric cancer survivors living in remote/rural/non-metropolitan communities during the COVID-19 pandemic. Future research views and avenues might help optimize virtual-care techniques, improve access and quality of care for CCSs living in non-metropolitan areas, and promote improved health outcomes for this vulnerable group.

## Figures and Tables

**Table 1:** Distribution of patients’ history by their location and type of visit.

		≥2 Late Effects	Total
Patients’ location	Non-metro area	130	437
	Metro area	307	
Type of visit	Virtual (phone and video)	160	437
	In person	277	

**Table 2:** Distribution of patients’ location by the type of visit.

Type of Visit	Location		Total	*p*-Value
	Non-Metro Area	Metro Area		
Virtual (phone and video)		137	221	<0.0001
In person	89	404	493	
Total	541	173	714	

## Data Availability

Not applicable.
